# Effects of single- or pair-housing on the welfare of shelter dogs: Behavioral and physiological indicators

**DOI:** 10.1371/journal.pone.0301137

**Published:** 2024-06-12

**Authors:** Grace Hecker, Katherine Martineau, Mariah Scheskie, Rhonda Hammerslough, Erica N. Feuerbacher

**Affiliations:** 1 Department of Animal and Poultry Sciences, Virginia Polytechnic and State University, Blacksburg, Virginia, United States of America; 2 Department of Anthrozoology, Carroll College, Helena, Montana, United States of America; 3 Humane Society of Western Montana, Missoula, Montana, United States of America; Universidade do Porto Instituto de Biologia Molecular e Celular, PORTUGAL

## Abstract

Dogs are often housed alone in shelter settings to reduce injury and disease spread. However, social isolation can be a stressor for dogs. Prior studies have suggested that cohousing can produce behavioral and physiological benefits. These studies have typically focused on laboratory dogs or shelter dogs that have been kenneled for several months. Thus, those results might not necessarily generalize to shelter dogs, many of which have shorter lengths of stay than those dogs studied to date, and might be cohoused soon after intake. In fact, being pair-housed could, in the short term, be more stressful as dogs have to navigate novel social situations in small spaces. We investigated the behavioral and physiological effects of single- or pair-housing shelter dogs, most of which had recently entered the shelter. We collected behavioral data on 61 dogs (30 single-housed; 31 pair-housed) daily across seven days; we also collected urine for cortisol:creatinine analysis on a subset (22 single-housed; 18 pair-housed) for eight days (each day of the seven-day study plus a baseline sample on Day 0, prior to dogs’ enrollment). We found pair-housed dogs engaged in three stress-related behaviors (lip licking, whining, and ears back) significantly less frequently than single-housed dogs. When we analyzed the change in urinary cortisol:creatinine (Days 1–7 values minus Day 0 value), we found that pair-housed dogs generally showed a greater decrease in cortisol:creatinine levels than single-housed dogs. Pair-housed dogs also had significantly shorter lengths of stay, but we did not detect any effect on dog-dog skills. Overall, we found well-matched pair-housing can have both proximate and ultimate welfare benefits for shelter dogs.

## Introduction

Nearly four million dogs pass through shelters in the United States each year [1]. As shelters have increased the percentage of dogs they adopt out, they are able to hold individual dogs longer while they wait for an adopter, such that dogs’ lengths of stay have increased [2]. Being able to hold dogs longer as they await adoption is a desirable community resource, however this brings up new sheltering challenges: ensuring the dogs remain medically and behaviorally healthy during their shelter stay. These are challenging objectives; shelters are stress-inducing environments for dogs, resulting in increased cortisol levels [3] and stress-related behaviors [4, 5] in shelter dogs. A variety of factors are potential stressors in shelters including loss of attachment figure for surrendered dogs [6], excessive noise [7–9], spatial restriction [4, 10, 11] and limited access to social interactions [4, 11]. These stressors can decrease overall wellbeing [12], produce undesirable behaviors [13] that might make adoption or retention in the adoptive home challenging, and potentially suppress shelter dog immune systems [2], making them more vulnerable to disease. Thus, identifying interventions that increase shelter dog welfare and decrease stress are crucial.

Despite being a social species [14], dogs are often housed alone in shelters with an aim to reduce disease transmission and possible injury from inter-dog conflict [15]. However, this social isolation has been identified as a potential stressor; supporting this possibility, shelter dogs that were given 30 min of access to social interaction and play with other dogs had lower cortisol levels and showed fewer stress-related behaviors [16] compared to dogs that stayed in their kennels. As such, pair-housing might be a useful intervention for improving shelter dog welfare.

Prior research has investigated both behavioral and physiological impacts of single- versus pair-housing. Social isolation was equally or more detrimental to kenneled laboratory beagles than spatial restriction [17]. Similarly, the dogs vocalized less, manipulated the cage less (chewed, pawed, or licked wire), and slept more when they were pair-housed than when housed alone [17], although other studies have found that single-housed dogs rested more than group-housed dogs [10]. Additionally, a higher percentage of single-housed dogs engaged in repetitive behavior [10].

Beerda et al. [4, 11] evaluated the effects of spatial and social restriction in laboratory beagles that had been previously cohoused. Dogs that had experienced nice weather when housed in a group outside showed more behaviors associated with stress and poor welfare [4], as well as increased salivary and urinary cortisol [11] when they were spatially and socially restricted indoors (but not in dogs that experienced bad weather when housed outside, suggesting a contrast effect between the previous and current situation). Of course, we cannot rule out the effect of indoor versus outdoor housing on the observed cortisol changes.

Much of the research into cohousing dogs comes from laboratory dogs which are raised in kennel situations and often co-housed with the same dogs for years (however see [17], in which laboratory beagles were newly placed into dyads); as such, results from laboratory dogs that have been purpose-bred and have had stable social systems in the kennels might not pertain to shelter dogs, for which the shelter might be a novel environment and very different from their prior living conditions. Grigg et al. [18] evaluated the effect of pair-housing on the behavior and hair cortisol levels of dogs that had been donated to the veterinary school (i.e., dogs that had not been reared or purpose bred as laboratory dogs). They found a trend of decreased barking and pacing in pair-housed dogs, and an overall decrease in hair cortisol levels compared to levels from when the dogs were single-housed, although not to a statistically significant level. Nevertheless, their research is suggestive that pair-housed dogs might have better welfare.

Still, as the dogs in the Grigg et al. [18] study had already been housed in the facility for at least six months, the data might not fully parallel most shelter dogs’ experience. Despite the more recent longer lengths of stay of shelter dogs [2], shelter dogs are often only in the shelter for a few days or weeks (mean 34.6 days in 2013 in two shelters in New York, United States) [19]. While, the addition of a kennelmate might reduce the stress experienced by shelter dogs and serve as a buffer for the other environmental stressors, the opposite is also possible: a novel kennelmate might add to dogs’ experienced stress because the dogs must now navigate social interactions with an unfamiliar dog in a small space, in addition to still being subjected to the other environmental stressors. Additionally, Grigg et al. [18] collected behavioral samples twice per week and sampled hair cortisol before and after pair housing, providing a more long-term view of the effects of pair-housing. Evaluating the effects of pair-housing on shelter dogs that have more recently entered the shelter and using a more frequent data sampling could help elucidate the shorter-term behavioral and physiological effects of being housed with a new kennelmate in a stress-inducing environment.

The goal of the current study was to assess the effect of single- and pair-housing on the behavior and physiology of shelter dogs during the first seven days of being pair-housed. To do this, we enrolled initially single-housed dogs and compared their daily in-kennel behavioral and physiological responses (urinary cortisol:creatinine) to those after they were first moved to a new kennel and pair-housed, or when they were moved to a new kennel and continued to be single-housed. We chose urinary cortisol:creatinine as our physiological measure as it has been a recommended noninvasive method for assessing physiological responses to stressors [20], has been used successfully with shelter dogs [e.g, 6, 11, 21, 22], and has a relatively short reflection period, such that our daily collections would allow us to investigate the shorter temporal dynamics of pair-housing.

We also assessed the dogs’ dog-dog skills on a standardized test at enrollment and again for any dogs that stayed for two weeks or more, and we measured their length of stay. Being kenneled with another dog could improve dog-dog social skills or at least prevent deterioration of skills into aggression. Additionally, beyond potentially improving behavior, being kenneled with another dog might make dogs more attractive to adopters by simply being co-housed, for example by being demonstrably dog-friendly or by making active behaviors appear more appropriate [see 23].

## Methods

All procedures were approved for ethical use of animals by the Carroll College Institutional Animal Care and Use Committee (Protocol #CC00008) and the Virginia Tech Institutional Animal Care and Use Committee (Protocol #18–073).

### General overview

We compared the behavioral and physiological responses of pair-housed dogs when they were first kenneled together in a new kennel to single-housed dogs that were moved to a new kennel by themselves. Prior to the experimental intervention, all dogs were housed singly. We collected the behavioral and physiological measures daily for seven days, to allow a finer-grained evaluation of the short-term effects of being housed with a new kennelmate.

We enrolled dogs by first testing them for dog-dog social skills using the Match Up II dog-dog subtest [24]. If a dog passed the subtest, it was assigned to being kenneled alone or with a companion for seven days. We collected four videos per day for seven days to analyze for in-kennel behaviors that might be indicative of stress [e.g., 15]. We also collected daily urine samples for cortisol:creatinine analysis for one baseline day (day of the dog-dog test) and the seven days the dogs were assigned to their experimental condition. The baseline collection occurred prior to the dog-dog test, and before dogs were moved to new kennels for the study (and before pair-housed dogs were assigned to pair-housing). For dogs that stayed at the shelter for at least 2 weeks, we re-assessed them on the dog-dog test ever 2 weeks to determine if there was any change in their dog-dog skills across time and if this was correlated with their housing type (single or pair). We also compared length of stay between dogs that were housed alone or with a companion to determine if housing status impacted adoption rates.

### Setting

**Fig 1:**
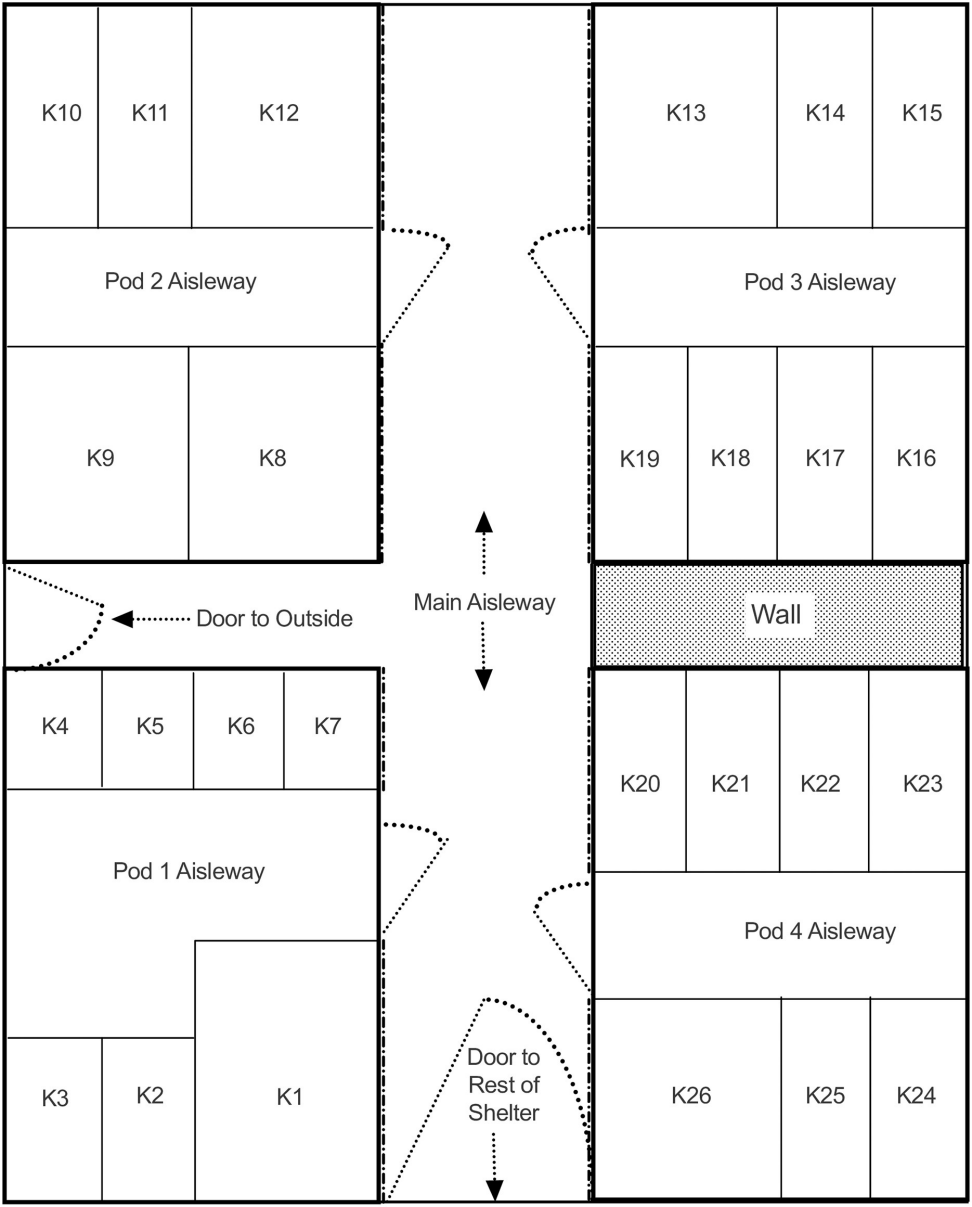
Schematic of main kennel area. Kennels are (labeled K1-K26) in the four pods. Not drawn to scale. The dashed lines along Kennels 1, 7, 8, 12, 13, 19, 20, and 26 indicate the windows of those end kennels that looked out onto the main aisleway.

We conducted the study at the Humane Society of Western Montana (HSWM), Missoula, MT in December 2017 and May 2018; approximately half of the dogs were enrolled in December and the other half were enrolled in May. Within each cohort, we balanced the number of single-housed dogs and the number of pair-housed dogs so that each housing type was equally represented in the two cohorts. HSWM is a limited-intake shelter with 26 kennels and two real life rooms (RLR). The shelter has four pods of kennels, each pod in its own room (Fig 1). There were two general kennel sizes: 1) large kennels (approximately 2.3 m x 2.7 m); and small kennels (approximately 1.2 x 2.0 m), and we considered them to be end kennels (kennels that were along the main aisleway) or internal kennels (all other kennels). All kennels had a front door that was opaque on the lower half and transparent plexiglass on the top half. End kennels in each pod had an additional full height transparent window approximately 20 cm across next to this front door. End kennels also had a half-window (bottom wall was opaque, window bottom approximately 1 m high) that opened onto the hallway connecting the pods. Thus, dogs in end kennels could see people walking down the main hallway, whereas dogs in internal kennels did not have this access. Additionally, the two real life rooms were similar in size to the large kennels and had windows that opened onto hallways or the lobby, similar to the large end kennels. Typically, large- and medium-side dog pairs were housed in the large end kennels or large internal kennels, and small dog pairs were housed in small-sized kennels. Individual dogs were typically housed in small kennels. One single-housed dog was kenneled in a large end kennel (Beefcake) and two dogs were housed in the RLR kennels (Neo and Sam). All of the kennels at HSWM were accessible to the public when the shelter was open.

### Subjects and selection

To be considered for the study, dogs had to be at least 6 months old. Eligible dogs were tested for dog sociability using the Match Up II dog-dog test [23, 24]. In this test, we selected a dog that we expected to be neutral based on staff recommendations and that was similar in size to the dog being tested. We followed the dog-dog subtest protocol. Two assistants walked the dogs past each other on leash approximately 1 m apart. If the target dog growled, bared its teeth or snapped, the test ended, and the dog was not included in the study. If the dog did not show any of these behaviors, the two assistants let the dogs approach and meet on leash, keeping the leashes as loose as possible and circling with the dogs to prevent leash tangles, for 30 s. The dog was enrolled in the study if 1) it did not show aggressive behaviors during this interaction and 2) it recovered from any fearful behaviors within the 30 s interaction period. As indicated in the Match Up II protocol, if the target dog showed extreme fear or aggression, it could be re-tested with another dog at the discretion of the experimenter.

We tested a total of 66 dogs in the Match-Up II dog-dog test. Three of these dogs were re-tested with a second dog and enrolled in the study based on their behavior with the second dog. Five dogs were not enrolled in the study due to displaying aggressive behaviors during the dog-dog test. Thus, we enrolled 61 dogs in the study. The demographics of the enrolled dogs are provided in S1 Table.

### Single- and pair-housing procedures

After completing the Match-Up II dog-dog test, dogs were pseudorandomly assigned to be housed alone (single-housed) or housed with a companion dog (pair-housed), with the caveat that we tried to balance breed types across groups as morphology is a primary determinant of adoption rate and length of stay [25, 26]. To this end, we tried to balance small dogs (less than 11.5 kg), bully-type dogs, and hound-type dogs across groups (see S2 Table for dog demographic distributions). After the Match-Up II test, dogs were introduced to another dog that, based on staff information, energy level, and size of the dog, we expected might be an appropriate kennel mate. Typically, these were other dogs that were being enrolled in the study at the same time, although sometimes these were dogs in the shelter that were not part of the study but were available to be pair-housed.

Prior to being tested for the study, all dogs were kenneled by themselves. After testing, all enrolled dogs were moved to a new kennel; dogs assigned to pair-housing were moved with their kennelmate to a new kennel and dogs assigned to single-housing were also moved to a new kennel to ensure an equivalent kenneling experience for all dogs. Thus, by the afternoon after the Match-Up II dog-dog test, all dogs had been moved to a new kennel.

All dogs stayed at the shelter for seven days after being enrolled in the study. Dogs were available for adoption during the study but did not go home with the adopter until the seven days were complete. For dogs that stayed longer than seven days, we worked to maintain their single- or pair-housing status until they were adopted. All single-housed dogs remained as single-housed until they were adopted. If the kennelmate of a pair-housed dog was adopted, we tried to find a compatible kennelmate for the remaining dog. For Goldie and Junior, they were kept as pair-housed after their initial kennelmate was adopted but by Week 4 there were not appropriate kennelmates for either (and Goldie was enrolled in May and Junior in December so it was not possible to pair them up with each other). Thus, no dogs participated in the study beyond 4 weeks.

Other than implementing pair-housing with dogs, we did not change any other procedures or systems the shelter had in place, including enrichment for dogs. Additionally, two dogs, Bacon and Gracie, were given crates part way through their stay as these were deemed beneficial by the staff.

### Behavioral data

Starting the day after the dogs were tested in the Match-Up II dog-dog test and moved to new kennels, we recorded four 1-min videos per day. The first video of each day was taken on a smartphone by a member of the research team standing in front of the kennel and recording the dog for 1 min to replicate the procedure by Protopopova and colleagues [27] and allowed us to assess dogs’ behavior when a human was standing in front of their kennel, as an adopter might. These first videos were taken within 10 min of the dog being returned to the kennel after being walked in the morning.

The other three videos of each day were collected by webcams attached near the top of the dogs’ kennels. The cords for the individual webcams were hung just inside the door of the individual pods. The team member opened the pod door to access the cords and pulled them into the hallway connecting the pods to connect the cords to computers for recording. Although gathering the cords typically did not interrupt the dogs’ behavior, we allowed 1 min to elapse before we began recording. During videoing, the team member remained behind brick walls so that they were not visually accessible to the dogs.

Webcam videos were collected half an hour before the shelter opened each day (12:30 pm Monday through Friday; 11:30 am Saturday and Sunday), and 1 and 3 hours after the shelter opened (2 pm and 4 pm Monday through Friday; 1 pm and 3 pm Saturday and Sunday). Thus, we collected data on the dogs’ behavior when a person was in front of their kennel (first morning video), remotely while the shelter was closed (12:30 pm/11:30 am video), and remotely while the shelter was open and potential adopters were walking through (2:00 pm/1:00 pm and 4:00 pm/3:00 pm videos).

#### Behavioral data analysis

We coded the in-kennel videos for 63 separate behaviors (see Table 1 for behaviors, behavioral definitions, and source of behavioral definitions of the analyzed behaviors that we report in the main manuscript; see S3 Table for the behaviors, behavioral definitions, and source of behavioral definitions for all behaviors we coded). Of these 63 behaviors, 54 were behaviors that both single- and pair-housed dogs could exhibit. Nine behaviors only pair-housed dogs could exhibit. Videos were analyzed by a research assistant who was trained in the behavioral definitions and was blind to the predicted outcomes of the intervention on these behaviors. Videos were scored in 5-sec intervals using partial interval recording such that if a behavior occurred any time during the 5 sec interval, the interval was scored as positive. We then calculated how many intervals each behavior occurred in as a measure of behavioral prevalence [27]. We had a second independent observer code 15% of the videos for interobserver agreement (IOA). We scored IOA by comparing interval by interval for all possible behaviors and calculated a percentage agreement for each video. Percentage agreement for individual video ranged from 70.37%-100% (mean agreement overall = 95.87%). Only *facing forward* and *facing away* had IOA in the 70% range in individual videos; all other behaviors reached at least 80% agreement in all videos that were double-coded.

**Table 1:** Behavioral definitions of analyzed behaviors. Name, definition, and source of definition for behaviors that qualified for analysis based on our frequency criterion. * indicates behaviors that we only coded for pair-housed dogs.

Name	Definition	Source
Body Position		
Front of kennel	Located between front of kennel and up to and including the midpoint of kennel. For end kennels, this also includes interacting with the side window (looking up at the window while next to it, or standing up and looking out the window).	[27]
Back of kennel	Located between back wall of kennel and up to, but not including, the midpoint of kennel	[27]
Lying down	Lying down with limbs either tucked under or placed in front of body	[27]
Sitting	Supported by two extended front legs and two flexed back legs	[27]
Standing	Supported upright with all four legs	[27]
Head up (lying down)	Lying down on ventral or side of body with limbs either tucked or placed in front of body without head making contact with arms, paws, bed, or floor	[5]
Head down (lying down)	Lying down on ventral or side of body with limbs either tucked or placed in front of body with head resting on limbs, paws, bed, or floor	[5]
Face Orientation & Movement		
Facing forward	Head is oriented such that an observer standing at the front of the kennel would be able to see more than the side profile of face. For end kennels, this should be considered from the perspective of the front of the kennel or from the side window.	Adapted from [27]
Facing away	Head is oriented such an observer standing at the front of the kennel would not be able to see more than the side profile of face. For end kennels, this should be considered from the perspective of the front of the kennel or from the side window.	Adapted from [27]
Gazing	Eye contact with the eyes of the observer (only for the 8:00 am videos)	[27]
Ears back	Ears folded against sides and/or back of head and having a flattened appearance	[27]
Lip licking	Extrudes portion of tongue and runs it over its lips	[28]
Tail Position		
Wagging tail	Tail moves perpendicularly to the dog’s body	[27]
Locomotion		
Moving forward	Distance between the dog and the front of the kennel is decreased (only code during 8:00 am videos)	Adapted from [27]
Moving away	Distance between the dog and the front of the kennel is increased (only code during 8:00 am videos)	Adapted from [27]
Standing on kennel	Both front paws make contact with the kennel and dog maintains position for >1 second (e.g., Dog standing to look out window)	Our definition
Jumping on kennel	Both front paws make contact with the kennel door that does not include lunging	[27]
Enclosure Contact/Exploration		
Sniffing	Muzzle/nose is oriented in a clearly observable direciton and motion of nostrils is observed	[27]
On bed	Has three or more paws on the raised dog bed. Any position (sitting, standing, or lying down)	Our definition
Vocalization		
Barking	Vocalization of very short duration and low frequency	[27]
Whining	A cyclic vocalization	[27]
Grooming and Maintenance		
Licking self	Oral contact with any part of body	[27]
Yawning	Opens mouth widely and inhales	[27]
Panting	Tongue exposed with audible and/or observable breathing	[27]
Social Interaction & Play		
Physical contact*	Dogs in physical contact with each other and not during a play or aggressive bout (e.g., sleeping or standing while in contact)	Our definition
Proximity*	Dogs not in physical contact but within one dog’s length of the largest dog of the pair for at least 2 seconds	Our definition
Single object play	Engagement with a toy or other object that involves tossing the toy, chewing or squeaking it, rolling it and chasing/pouncing on it. Object could be bedding, but excludes chewing bedding.	Mehrkam (personal communication)

We omitted from statistical analysis behaviors that occurred in fewer than 5% of videos. This resulted in 36 behaviors being omitted from analysis. For the analyzed behaviors, we calculated the mean number of intervals each dog engaged in each behavior each day and analyzed that. Because the data were not normally distributed, we used an aligned rank transformation [29, 30] in R Studio and employed a linear model to analyze the transformed data. Housing type (single- or pair-housed) and day were entered as fixed effects, and dog was entered as a random effect. We analyzed the behavioral data for the main effects of housing type (single- vs pair-housing) and day, as well as housing type x day interactions. For those behaviors that were only able to be exhibited by pair-housed dogs, we only analyzed those behaviors for the main effect of day, such that day was a fixed effect and dog a random effect in the model.

For any main effects of day or interaction effects we detected, we conducted post-hoc tests using an aligned rank tool for multifactor contrast [31] in R Studio. Our results were corrected for multiple comparisons using the Tukey method for family comparisons.

### Cortisol:creatinine levels

We collected urine for cortisol:creatinine (C:C) analysis on a subset of the dogs (22 single- and 18 pair-housed dogs). The first collection occurred on the morning prior to their Match-Up II dog-dog test and served as a baseline value for each dog. We continued to collect urine every morning for the next seven days, to capture dogs’ physiological response to being housed alone or with a companion. We collected urine following the technique of Gunter et al. [22]. We took dogs out of their kennels and leash walked them in an approved area. We used an Olympic Clean-Catch^™^ plastic tray taped to a “Pickup and Reach” tool (Harbor Freight, Calabasas, CA), which was approximately 91 cm long. Trays were rinsed with water before each collection. Immediately after collection, urine samples were poured into 10 mL plastic conical centrifuge tubes with screw caps for storage and shipment. All but one sample were collected between 7:00 am and 9:30 am, with that sample collected at 9:55 am. This sampling window has been found to be effective for detecting C:C differences in shelter dogs [22]. Dogs that we did not collect urine from also received a morning walk during the same time frame as the dogs that were part of the urine collection subset.

Samples were immediately placed in a freezer. They remained frozen until they were shipped overnight for analysis. Urine was analyzed for cortisol:creatine by Applied Biosciences (Wellborn, TX). Samples were analyzed within 1 month of collection. Cortisol was analyzed by radioimmunoassay (MP BioMedicals (07–221106) and counted on an ISO 20/20 Gamma Counter for 1 min. Creatinine was analyzed using ELISA (reagents manufactured by Arbor Assays; Urine Creatinine Detection [K002-H5]). The Optical Density (OD) was read at 490 nm on a plate reader (Vmax from Molecular Devices) and analyzed using Softmax Pro 5.4.

#### Cortisol:creatinine data analysis

We conducted a multiple linear regression analysis on Day 0 C:C levels with backward elimination to determine whether C:C levels could be predicted from variables related to the dog (weight, age, sex, neuter type, days in shelter, and intake type). Using the C:C from Days 1–7, we calculated the change in C:C (DC:C) by subtracting the C:C value of each of the Days 1–7 from the C:C value of Day 0. Because these data were not normally distributed, we used an aligned rank transformation [29, 30] in R Studio and employed a linear model to analyze the transformed data. Housing type (single or pair-housed) and day were entered as fixed effects, and dog was entered as a random effect. We conducted post-hoc Mann-Whitney U tests on the raw DC:C data.

### Length of stay

We calculated the length of stay (LOS) for dogs to determine if being housed alone or with a companion impacted adoption rates. This was calculated as the number of days between when the dog entered the shelter and when it was adopted. If the dog was not able to go home when potential adopters met with the dog (i.e., it was during the seven days of the study or the dog was not yet altered), we counted the adoption date as the date a hold was placed on the dog that turned into an adoption. We excluded dogs that had been there for more than one day prior to their enrollment in the study as potential adopters could have seen the dog before they were kenneled in the experimental designation. We additionally excluded dogs who changed from pair- to single-housed before being adopted and dogs who went into foster. Thus, 27 dogs were excluded from this analysis.

#### Length of stay data analysis

We conducted a multiple linear regression analysis with backward elimination to determine whether LOS could be predicted from variables related to the dog (weight, age, sex, intake type, breed type) or where it was housed (kennel type: small end kennel, large end kennel, small internal kennel, large internal kennel, or real-life room) and how it was housed in the shelter (housing type: single- or pair-housed).

### Dog-dog test

We conducted a dog-dog test on Day 0 (before the dogs were assigned to their experimental group) for all dogs (*n =* 61) and again at Week 2 for all dogs that remained in the shelter and were in their experimentally-assigned housing groups (*n* = 8.). Day 0 and Week 2 tests were conducted the same way. We did not conduct any other assessments after Week 2 because at Week 4, only Goldie and Junior remained at the shelter, both of which were pair-housed dogs and had been recently switched to single-housing because no appropriate kennelmates were available.

We video-recorded all dog-dog tests and scored the videos to calculate the dog’s exact score on the dog-dog test for comparison to any future dog-dog tests. To ensure that more subtle behaviors that might be challenging to detect on video were identified, the dog handlers called out vocally any behaviors included in the dog-dog test rubric that they noticed while they conducted the dog-dog test.

#### Dog-dog test data analysis

For the eight dogs that completed two dog-dog tests, we scored their first and second dog-dog tests in accordance with the Match Up II scoring sheet and compared their scores on the first and second tests to determine if housing type impacted dog-dog skills. Each dog received a score on five personality dimensions: *Friendliness*, *Fearfulness*, *Excitability*, *Aggressiveness*, and *Playfulness*, which were then summed to produce their overall score. Because the overall scores were not normally distributed, we used an aligned rank transformation [29, 30] in R Studio and employed a linear model to analyze the transformed data. Housing type (single or pair-housed) and test number were entered as fixed effects, and dog was entered as a random effect.

## Results

During the study, two pairs of dogs were separated for showing aggression (Wilson and Gracie; Max and Lucy). Wilson, Gracie and Max were each kenneled afterwards as single dogs. Lucy was paired with another dog (Devin) and remained in the study as a pair-housed dog. Thus, we had a total of 30 single-housed dogs and 31 pair-housed dogs. The aggression that resulted in the decision to separate the pairs all occurred within 36 hours of the dogs being kenneled together and no injuries were incurred from these incidents or in any other dogs during the study.

### Behavioral data

From the 61 enrolled dogs, we collected 1,662 1-min videos of the dogs. Forty-six 1-min videos were lost due to experimenter error, becoming corrupted, or a dog being sent home for adoption before we collected the last video. We report below only on those behaviors we analyzed and for which 1) we found statistically significant differences or 2) were pair-housed behaviors that reached our analysis threshold. Table 2 lists the statistical results for main effects of day and housing type, and their interaction. In the text, we organized our results by whether the behavior had only main effects of housing type or an interaction of housing type x day (there were no behaviors that had *only* a main effect of day). However, in Table 2 and figures, we report on and graph all behaviors showing main effects, including those that are discussed in the interaction section of results. The statistical results for all analyses we conducted can be found in the archived dataset.

**Table 2:** Behaviors with a significant main effect or interaction.

Behavior	*F-*statistic	*P-value*	ηp ^2^
Behaviors with a significant main effect of housing status			
Gazing	17.52 (1, 54)	.00001	.245
Lip Licking	9.28 (1, 59)	.003	.136
Standing on Kennel	4.42 (1, 59)	.040	.070
Ears Back	4.13 (1, 59)	.047	.076
Whining	53.84 (1, 59)	7.252e-10	.477
Single Object Play	17.75 (1, 59)	8.736e-05	.231
Behaviors with a significant main effect of day			
Ears Back	3.71 (6, 354)	.0013	.063
Whining	4.64 (6, 354)	.0001	.073
Single Object Play	2.95 (6, 354)	.008	.048
Behaviors with a significant interaction of day x housing status			
Moving forward	2.61 (6, 346)	.017	.046
Ears Back	7.06 (6, 354)	4.040e-05	.107
Whining	3.72 (6, 354)	.001	.059
Single Object Play	5.69 (6, 354)	1.149e-05	.088

#### Behaviors with significant main effect of housing type only

**Fig 2:**
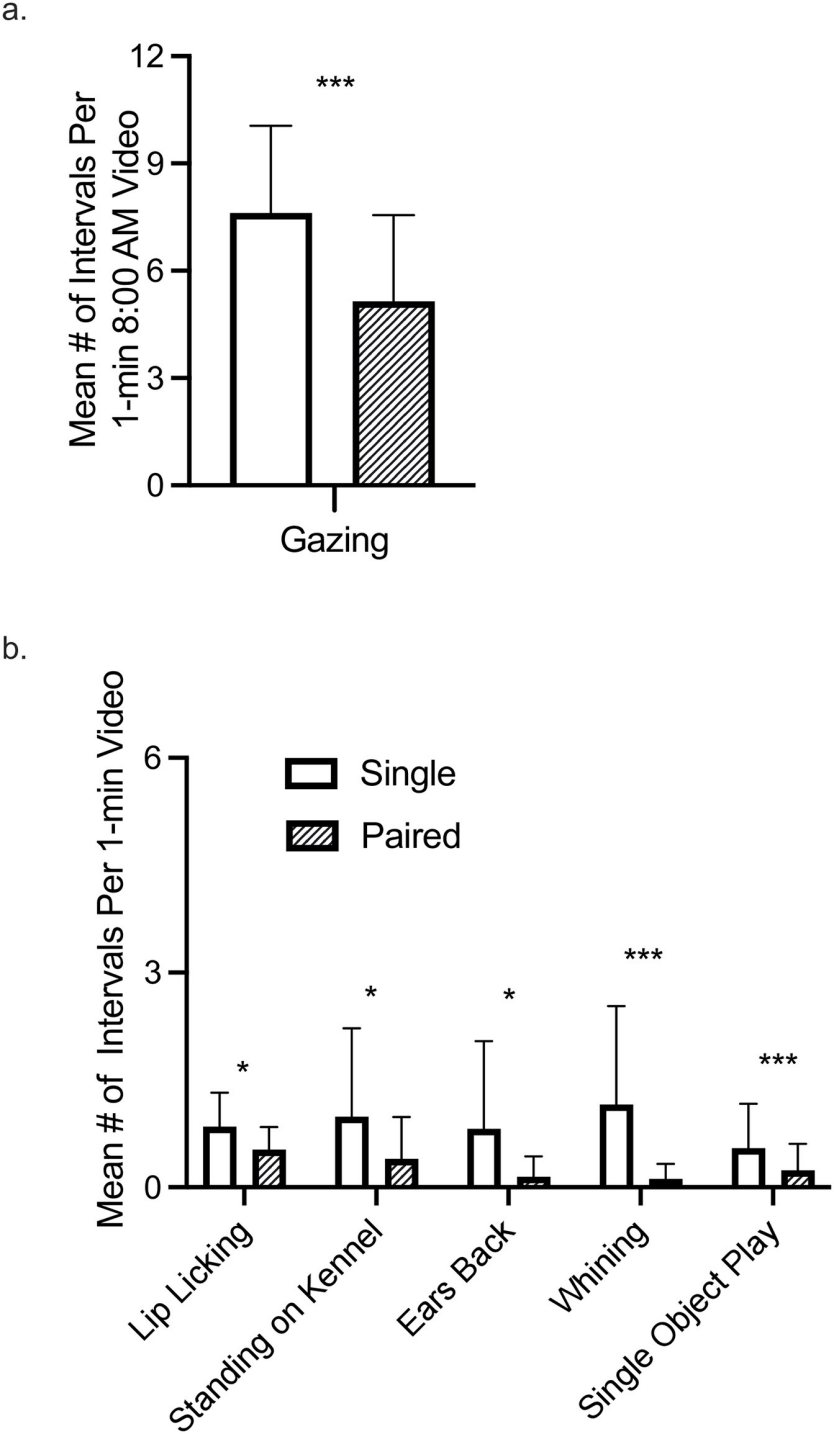
Significant main effect of housing type. Behaviors that significantly differed between single- and pair-house dogs. *a*. Gazing behavior that was only recorded during the 8:00 am videos. *b*. Behaviors that were coded for all videos recorded. * *p* < .05, ** *p* < .01, *** *p* < .001.

*Gazing*. *Gazing* was only analyzed from the 8:00 AM videos. We found a significant main effect of housing type (Fig 2a; Table 2) with single-housed dogs *gazing* a significantly greater mean number of intervals per day than pair-housed dogs.

*Lip licking*. We found a significant main effect of housing type (Fig 2b; Table 2), with single-housed dogs engaging in *lip licking* a significantly greater mean number of intervals per day than pair-housed dogs.

*Standing on kennel*. We found a significant main effect of housing type (Fig 2b; Table 2), with single-housed dogs engaging in *standing on kennel* a significantly greater mean number of intervals per day than pair-housed dogs.

#### Behaviors with significant day x housing type interaction

**Fig 3:**
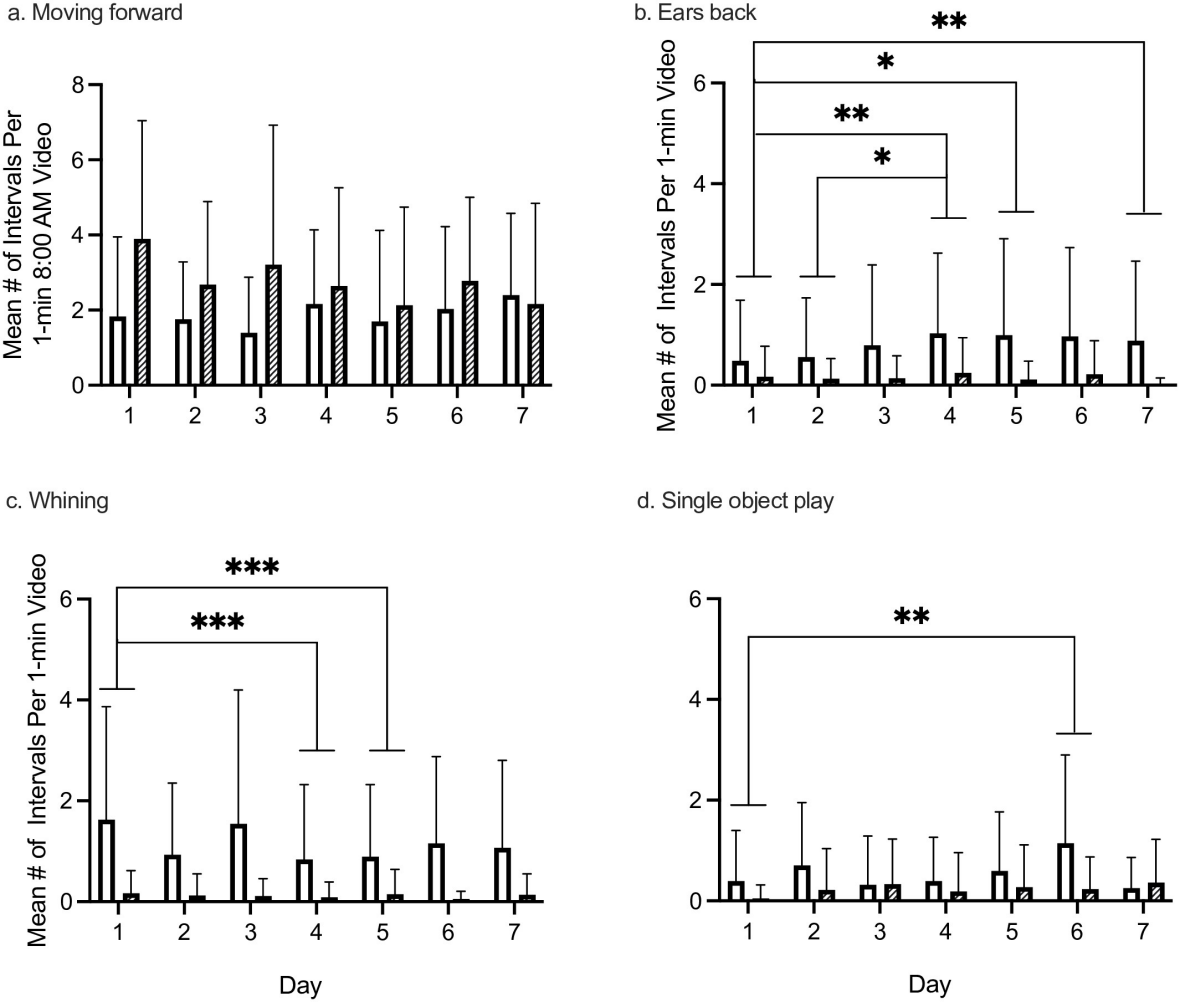
Significant interactions and main effects of day. Behaviors that had a significant interaction between day x housing type. We found no significant interaction differences when we conducted post hoc tests for interaction comparisons. Because three of these behaviors were also the only behaviors that had a significant main effect of day, we have indicated on this graph those significant day effects (denoted by a horizontal line over both single- and pair-housed bars for each day that was different). * *p* < .05, ** *p* < .01, *** *p* < .001.

*Moving forward*. *Moving forward* was analyzed only from the 8:00 AM videos. While we found no significant main effects of housing type or day, we did find a significant interaction between housing type x day (Fig 3a; Table 2). However, post hoc tests for interaction effects did not reveal any significant differences.

*Ears back*. We found a significant main effect of housing type (Fig 2b; Table 2) with single-housed dogs engaging in *ears back* a significantly greater mean number of intervals per day than pair-housed dogs. We also found a significant effect of day (Fig 3b, Table 2). Post hoc tests revealed that dogs exhibited ears back in significantly fewer mean number of intervals on Day 1 compared to Day 4, Day 5, and Day 7; and dogs exhibited ears back in significantly fewer mean number of intervals on Day 2 compared to Day 4. We also found a significant interaction of housing type x day (Fig 3b; Table 2). However, post hoc tests revealed no significant differences.

*Whining*. We found a significant main effect of housing type (Fig 2b; Table 2), with single-housed dogs engaging in *whining* a significantly greater mean number of intervals per day than pair-housed dogs. We also found a significant effect of day (Fig 3c; Table 2). Post hoc tests for main effect of day revealed that dogs engaged in whining a significantly greater mean number of intervals on Day 1 than Day 4 and Day 6. We also found a significant interaction of housing type x day (Fig 3c; Table 2). However, post hoc tests revealed no significant differences.

*Single object play*. We found a significant main effect of housing type (Fig 2b; Table 2), with single-housed dogs engaging in *single object play* a significantly higher mean number of intervals per day than pair-housed dogs. We also found a significant effect of day (Fig 3d; Table 2). Post hoc tests for main effect of day revealed that dogs engaged in *single object play* for a significantly greater mean number of intervals per day on Day 6 compared to Day 1, with a trend towards dogs engaging in significantly greater mean number of intervals per day on Day 6 than Day 3 (*p* = .0504) and Day 4 (*p* = .0517). We also found a significant interaction of housing type x day (Fig 3d; Table 2). However, post hoc tests revealed no significant differences.

#### Pair-housed dog behaviors

We found no statistically significant differences across housing type or day in the number of intervals per 1-min video pair-housed dogs engaged in physical contact (*M =* 1.53, SD = 3.46), or proximity (*M =* 8.01, SD = 4.85).

### Cortisol:creatinine levels

**Fig 4:**
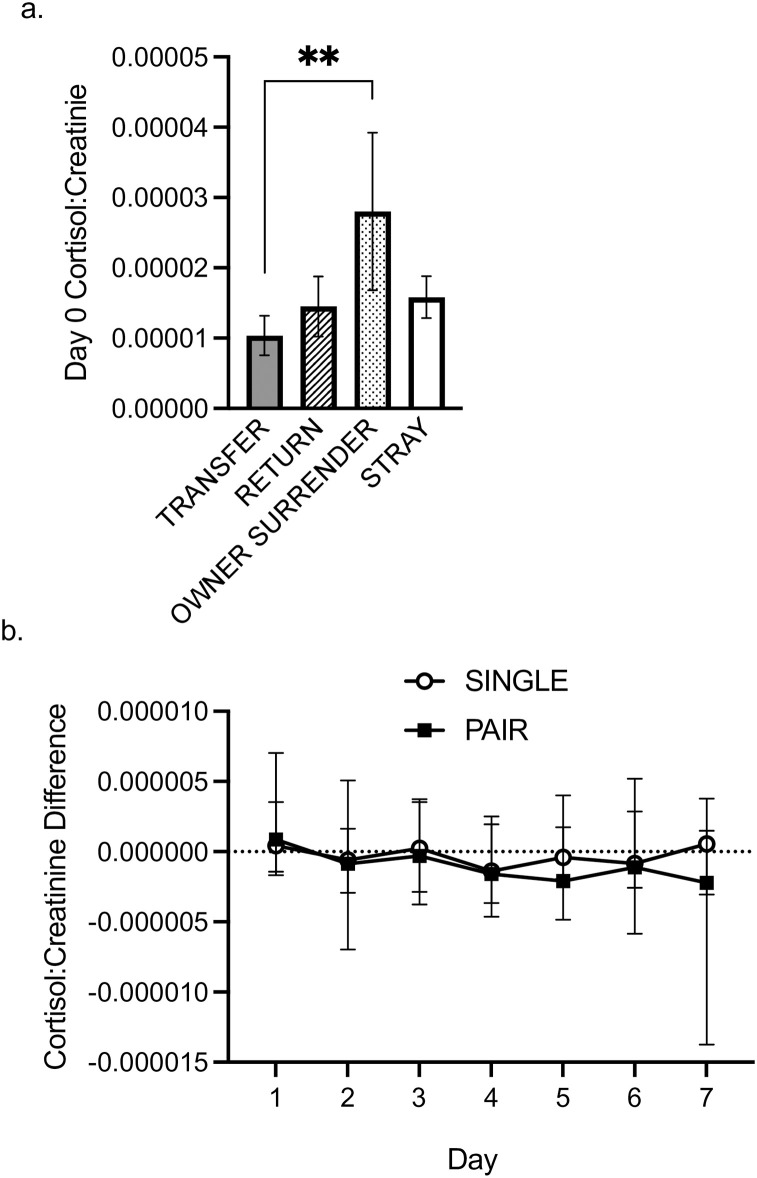
Urinary cortisol:creatinine. *a*. Day 0 urinary C:C levels for each intake type. *b*. Change in urinary C:C levels as compared to Day 0 in single- and pair-house dogs. ** *p* < .01.

We collected urine on 22 single-housed and 18 pair-housed dogs for eight days. From the multiple linear regression analysis on Day 0 C:C levels, only sex and intake type remained in the equation. Together they accounted for 10% of the variability in Day 0 C:C levels, *F* (2, 37) = 2.12, *p* = .134. The coefficient for intake type was significant (ß = 6.331 x 10^−6^, *t =* 2.059, *p* = .047). A post hoc Fisher’s least significant difference test on log transformed Day 0 C:C revealed that owner surrender dogs had significantly higher C:C levels than transfer dogs (Fig 4a; *p* = .043). No other differences between intake types were significantly different (next smallest *p* = .14). An independent samples *t*-test on the log transformed Day 0 C:C also revealed there was no significant difference between the single- or pair-housed dogs at Day 0 (*t* = -1.244, *p* = .22).

For the change in cortisol:creatinine levels (DC:C), there were no statistically significant main effects of housing (single or pair) or day, largest *F*(1, 228) = 1.223, *p =* .30, ηp ^2^ = .031). There was a statistically significant interaction between housing x day *F*(6, 228) = 2.169, *p* = .046, ηp ^2^ = .054 (Fig 4b). However, post-hoc Mann-Whitney U tests on the raw DC:C data did not reveal any statistically significant differences (smallest *p =* .11 for single- vs. pair-housed Day 7).

### Length of stay

After excluding dogs that 1) had been at the shelter for at least 1 day prior to being enrolled in the study, 2) changed from pair- to single-housed before being adopted or 3) were transferred to foster before being adopted, 14 single-housed dogs and 20 pair-housed dogs remained for use in the analysis on length of stay. In the best model of the multiple linear regression (identified by largest adjusted R^2^), weight, intake type, and housing type remained, *F* (3,30) = 3.78, *p* < .05. Only two of these coefficients were statistically significant: housing type (ß = -4.56, *t =* -2.139, *p* < .05); and weight (ß = .11, *t =* 2.463, *p* < .05). Dogs in pair-housing had shorter LOS (*M* = 6.60 days, SD = 5.43) than single-housed dogs (*M* = 10.78 days, SD = 7.89). In terms of weight, with every 10 kg increase in weight, LOS increased by 1 day.

### Dog-dog test

Eight dogs remained at the shelter long enough to have a second dog-dog test conducted. Six of these dogs were single-housed and two were pair-housed (Table 3). There were no statistically significant main effects of housing (single or pair) or day, or interactions, largest *F*(1,6) = 0.225, *p* = .63, ηp ^2^ = .041).

**Table 3:** Dog-dog test scores. Match Up II dog-dog test scores for the eight dogs that remained for at least two weeks. The Day 1 test was conducted on the day of their enrollment; Day 2 test was conducted 14 days later.

**Single-Housed Dogs**							
Dog	Test Day	Friendly	Fearful	Excited	Aggressive	Playful	**Total**
Bacon	Day 1	2	2	0	0	0	**4**
	Day 2	3	0	1	0	2	**6**
Beefcake	Day 1	4	0	2	1	0	**7**
	Day 2	3	0	2	1	0	**6**
Kira	Day 1	3	0	0	1	0	**4**
	Day 2	3	0	2	0	0	**5**
Mellow Yellow	Day 1	3	3	0	1	0	**7**
	Day 2	3	0	0	1	0	**4**
Sadie	Day 1	3	0	0	0	0	**3**
	Day 2	3	0	0	0	0	**3**
Wilson	Day 1	3	0	0	0	0	**3**
	Day 2	3	0	0	0	2	**5**
**Pair-Housed Dogs**							
Dog	Test Day	Friendly	Fearful	Excited	Aggressive	Playful	**Total**
Bobo	Day 1	3	0	0	0	0	**3**
	Day 2	3	0	0	0	0	**3**
Goldie	Day 1	3	1	0	0	0	**4**
	Day 2	3	0	2	0	0	**5**

## Discussion

We investigated the impact of pair-housing compatible dogs on their behavior, C:C values, length of stay (LOS), and scores on a dog-dog test. We found benefits of pair-housing for dogs in several of our experimental measures, including reduced mean number of intervals in which dogs exhibited three stress-related behaviors, a significant interaction for changes in C:C levels, with pair-housed dogs generally showing a greater reduction in C:C levels compared to baseline than single-housed dogs, and a reduction of length of stay for pair-housed dogs. All of these point to the welfare benefits of pair-housing for shelter dogs.

We found that single-housed dogs engaged in higher frequencies of three stress-related behaviors (lip licking, whining, and ears back) than pair-housed dogs. Prior research has demonstrated all three of these behaviors correlate with increased stress. Lip licking has been associated with chronic and acute stressors [4, 32–35] and Hennessy [12] noted that lip licking was one of the behaviors most consistently associated with stress. Increased vocalization has also been associated with chronic stressors in dogs [4], and ears back positively correlated with increased heart rate during a standard veterinary exam [36], and occurred after dogs experienced electric shock, loud noise, or an umbrella opening [33]. Together, these three behaviors suggest that pair-housed dogs experienced less stress than single-housed dogs, which aligns with results from other studies investigating the effects of cohousing [4, 17, 18, 37].

Both whining and ears back also had a significant main effect of day. Dogs engaged in whining a significantly greater number of mean intervals on Day 1 compared to later days. This could be an outcome of having been moved to a new kennel the day before, potentially increasing their stress. Supporting this, the change in cortisol levels (DC:C) for both single- and pair-housed was positive on Day 1, meaning that it was higher than their Day 0 C:C levels. On the other hand, ears back seemed to increase across days, although the increase was small. Since prior research has shown dogs might begin to habituate to the shelter around Day 4 [38], the increase in ears back, a stress- and fear-related behavior, is surprising, especially given the changes in whining that we noted above, which suggested a reduction in stress.

Other than the three stress-related behaviors discussed earlier that differed between single- and pair-housed dogs, we observed generally low frequencies of other behaviors that have previously been observed in stress-inducing settings, including when dogs were housed alone, such as repetitive, fear-related, or undesirable oral behaviors (Repetitive behaviors: [10, 13, 17]; Fear-related behaviors: [4, 33, 34]; Undesirable oral behavior: [4, 13]). The low frequencies of these other stress-related behaviors might speak to the relatively high level of enrichment HSWM provided all dogs, including a high level of human interaction, which has been demonstrated to be an effective stress-reduction intervention [e.g., 39, 40], and an in-kennel enrichment program.

The three other behaviors that we detected significant differences between single- and pair-housed dogs were *single-object play*, *standing on kennel* and *gazing*. For all of these, single-housed dogs engaged in these behaviors a significantly greater mean number of intervals; as such, we suspect that these might be linked to a lack of enrichment in single-housed dogs. For example, it is possible that single-housed dogs engaged in more single-object play because they lacked the enrichment of the other dog. We observed dog-dog play and pair-object in the pair-housed dogs, which likely competed with single-object play. We also observed more *gazing* behavior and *standing on kennel* in single-housed dogs. Both of these behaviors are ones in which the dog’s attention was typically oriented outside of the kennel (even though the behavioral definition of *standing on kennel* did not require this, most dogs were looking out a visual access point when they were coded as *standing on kennel*). Similar to the single-object play, it is possible that the kennelmate provided enrichment and competed with gazing at the experimenter or standing on the kennel. In fact, increased *standing on kennel* occurred in single-housed dogs despite the fact that the large end kennels where most pair-housed dogs were housed had windows that looked out on the main hallway. If dogs stood to look out those side windows, we counted this as standing on kennel. Thus, even when pair-housed dogs had likely more opportunity to stand on kennel we saw more standing on kennel in single-housed dogs.

We also detected a significant interaction for *moving forward*, with pair-housed dogs engaging in this behavior a greater number of mean intervals on all days except Day 7. It is unclear what might be the cause of this behavior is; there was no significant difference between single- or pair-housed dogs in terms of the mean number of intervals they were in the back of the kennel. Thus, it is not likely that pair-housed dogs were more often at the back of the kennel and thus had more opportunity to approach the front when the experimenter was videorecording them (*moving forward* was only coded from the 8:00 am videos). It is possible that if one pair-housed dog moved forward, the other was more likely to move forward due to social facilitation, and thus we observed more intervals in which pair-housed dogs approached. Nevertheless, the cause and the implications of this difference are unclear.

Our DC:C ratios point to some, but not conclusive, evidence that pair-housing dogs lower C:C levels more than singly-housing dogs. Overall, DC:C ratios did not significantly differ between single- and pair-housed dogs, which parallel results reported by Grigg et al. [18]. In that study, while the percent decrease in hair cortisol was greater for pair-housed dogs, it did not reach statistical significance [18]. However, when we investigated the interaction between housing type x day, we did find a significant interaction with a nearly medium effect size. While we were not able to identify differences in post hoc analysis, it is notable that pair-housed dogs showed a greater decrease in C:C values as compared to Day 0 than the single-housed dogs on all days of the experimental period except Day 1, and DC:C values for pair-housed dogs were also negative on all days of the experimental period except Day 1, whereas single-housed dogs showed positive DC:C values on three out of seven days, including on the last day. While we had a larger sample size than the most similar shelter dog study [18] an even larger sample might be warranted to effectively detect interaction effects in post hoc tests.

We also detected an interesting effect when analyzing our Day 0 C:C: owner surrender dogs had higher C:C values than transfer dogs, which parallels prior results in which owner surrender dogs showed increasing cortisol levels over time, while dogs that had come in as returns or strays showed lower levels [6]. Being separated from an attachment figure might function as an additional stressor. The result that transfer dogs showed lower C:C than the other groups, although only statistically different from owned dogs, is also curious. The majority of the transfer dogs were dogs that were flown on a plane from states south of Montana; the other transfer dogs were driven from shelters several hours away. Transport has been shown to increase cortisol levels in dogs including ground transport [32, 41, 42] as well as air transport [41]. Since the stress and welfare effects of current housing can be impacted by prior housing [11], it is possible that the shelter was less stressful than transport and this was reflected in transfer dogs’ C:C values.

Interestingly, on Day 7 our data showed the greatest difference between the single- and pair-housed DC:C levels, suggesting that a longer experimental period might have revealed statistically significant differences if that trend continued. Also notable is that both single- and pair-housed dogs showed a positive DC:C on Day 1. All dogs in our study were moved to new kennels on Day 0 such that this increase in C:C on day 1 might suggest that changing kennels is a mild stressor for dogs, regardless of what type of housing they were being moved into, and has potential implications for sheltering practices as dogs are often moved to new kennels as they make their way through shelter systems.

The effect of pair-housing dogs was also apparent in dogs’ LOS, with pair-housed dogs having a shorter LOS by 4 days on average compared to single-housed dogs. Our results parallel those from Mertens and Unshelm [37] in which pair-housed dogs were adopted, on average, 7 days sooner than single-housed dogs. Given that many potential adopters might already have a resident dog, or would like to engage in social activities with their dog, clearly exhibiting that a dog can successfully interact with other dogs might highlight those dogs as being good possible matches. Additionally, in our study, every 10 kg of weight added 1 day to the LOS, which aligns with prior research showing small dogs have shorter LOS [19, 25, 27, 43].

Although we did not see an effect of pair-housing dogs on changing dog social skills, either improving or worsening, when we conducted repeated dog-dog tests, we would caution against generalizing this result. First, we had few dogs that remained in the shelter for two weeks to test this question, and none that remained for a four-week test, such that the effects might become more apparent after longer time periods. Additionally, HSWM already provided a high level of dog-dog enrichment for their dogs, including dogs going to outside pens in pairs or groups. Thus, it is possible that in a shelter that provided no extra dog-dog interaction, co-housing would show an effect of maintaining or even improving dog-dog interaction skills.

## Conclusions

Our results add to the evidence that pair-housing compatible dogs could benefit both their proximate welfare, as evidenced by their behavioral and physiological responses, and their ultimate welfare, as evidenced by shorter lengths of stay, making this a potentially impactful intervention for shelter dogs. Our study expands prior research into a more naturalistic shelter population: dogs that are housed short term and are often paired with another dog soon after their entry into the shelter (mean number of days in the shelter at time of enrollment: single-housed dogs = 5.7, pair-housed dogs = 3.3). Because concerns over aggression is one of the main reasons shelters do not pair-house dogs, it is worth noting that, similar to other cohousing studies [18, 37], we had no injuries from pair-housing dogs, very low rates of agonistic behaviors (all occurred in less than 0.2% of intervals), and only had two pairs that we separated because of non-injurious aggression. Both of these occurred within the first 36 hours of cohousing the dogs, suggesting closer monitoring during the first few days is warranted.

Not only did we see low rates of aggression, we saw affiliative behaviors in the pair-housed dogs. We observed high levels of proximity, suggesting that dogs might choose to be near their kennelmate, although this could also be a product of small kennel size [17]. We also observed that in nearly 10% of the intervals, dogs were in physical contact with one another (excluding play or aggression) such that this suggests dogs might find comfort in having a kennelmate. In fact, while we observed an overall high usage of the raised kennel beds provided (mean of nearly 50% of observed intervals), we anecdotally noted that several pairs of dogs chose to share a bed, despite two beds being available for them. We also observed play initiation and dog-dog play in pair-housed dogs, with 14 pair-housed dogs engaging in dog-dog play. Providing dogs with opportunities to play have been associated with lower stress behaviors [16]. Of course, we urge judiciousness when pair-housing dogs; we assessed all the dogs using the Match Up II dog-dog subtest, matched enrolled dogs, and staff monitored them throughout the day, especially during the first days of cohousing. With this in mind, the benefits we noted of pair-housing dogs likely has limits and housing more dogs in a kennel is not necessarily better. Kenneling dogs with multiple novel dogs might compromise their welfare, as they are more limited in their ability to avoid each other, and making appropriate matches for all dogs in the kennel becomes unlikely.

Finally, there are many avenues for future research. Larger sample sizes could allow for detection of statistically significant post hoc results those behaviors for which we detected a significant interaction, but no significant post hoc results. Second, we saw some evidence that the single- and pair-housed dogs’ change in C:C were continuing to diverge at Day 7. Evaluating the effects of pair-housing for an even longer period of time would further elucidate the effects of pair-housing on welfare. Third, evaluating the effects of changing kennelmates would be beneficial, as shelter dogs are regularly adopted, transferred, and euthanized, and new dogs enter the shelter. Fourth, as we conducted this in a working shelter, we, just as shelters are, were constrained by the physical properties of the shelter, such that dogs were housed in different types of kennels. Generally, we maintained a constant dog:kennel space ratio, with pair-housed medium and large dogs being kenneled in larger kennels, although we did have one large dog kenneled alone in a large kennel, and some small dog pairs were kenneled together in small kennels. While Hetts et al. [17] found that social status (single- or pair-housed) was a more important factor than kennel size for dog behavior and welfare, studies that can ensure constant kennel size across all dogs or a constant dog:kennel space ratio across all dogs would be useful. Lastly, the shelter where we conducted this study had a robust enrichment program in place, including allowing dogs to socialize in small groups in large outdoor pens during cleaning, and a pod design that might have helped limit other stressors such as excessive noise and other unpredictable stimuli. We hope future research will replicate this study at other shelters with different housing and husbandry practices, especially shelters that do not already provide opportunities for regular conspecific social interaction, as the benefits of cohousing dogs might become even more apparent in those shelters.

## Supporting information

S1 Table Demographic data of the dogs in Experiment 1. Age is reported in years (y) and months (m). Sex: F is female, M is male, S is spayed, N is neutered, U is unaltered. Under breeds the predominant breed type is listed. We used breed type categorization based on that described by Protopopova et al. [27]. * denotes dogs that we collected urine from for C:C analysis. Days in shelter at start of study indicates how long the dog had been in the shelter at the time of their enrollment in the study.
(DOCX)

S2 Table Distribution across groups of different dog demographic dimensions. (DOCX)

S3 Table Name, definition, and source of definition for all behaviors coded in the videos. (DOCX)
