# Supplementary material for: Effects of single- or pair-housing on the welfare of shelter dogs: Behavioral and physiological indicators
Source: PLoS One. 2024 Jun 12;19(6):e0301137. doi: 10.1371/journal.pone.0301137 (PMC11168620; doi:10.1371/journal.pone.0301137)
Supplement: S1 Table — Age is reported in years (y) and months (m). Sex: F is female, M is male, S is spayed, N is neutered, U is unaltered. Under breeds the predominant breed type is listed. We used breed type categorization based on that described by Protopopova et al. [27]. * denotes dogs that we collected urine from for C:C analysis. Days in shelter at start of study indicates how long the dog had been in the shelter at the time of their enrollment in the study. (DOCX) [file pone.0301137.s001.docx]

**S1 Table. Dog Demographics**

| Single-Housed Dogs | | | | | |
| --- | --- | --- | --- | --- | --- |
| Dog | Breed | Age | Sex | Source | Days In Shelter at Start of Study |
| Bacon* | Bully-type | 3y | NM | Transfer | 5 |
| Beefcake* | Bully-type | 2y | NM | Transfer | 1 |
| Bruin* | Working | 3y 1m | NM | Surrender | 3 |
| Chief* | Working | 1y | NM | Transfer | 3 |
| Coconut* | Herding | 1y | UF | Stray | 1 |
| Cosmo | Lap | 6y | NM | Surrender | 1 |
| Daisy* | Sporting | 7y | SF | Surrender | 1 |
| Doug* | Lap | 3y | UM | Surrender | 1 |
| Ducky | Herding | 10m | UF | Transfer | 1 |
| Frida* | Working | 5y | SF | Surrender | 16 |
| Ginger* | Bully-type | 6m | SF | Return | 13 |
| Gracie | Sporting | 1y 1m | SF | Return | 25 |
| Jay Z* | Lap | 6y | NM | Transfer | 1 |
| Jessie* | Lap | 3y | NM | Transfer | 1 |
| Karl* | Working | 10m | NM | Surrender | 7 |
| Katie* | Herding | 10y | SF | Transfer | 1 |
| Kira* | Sporting | 5y | SF | Return | 1 |
| Max | Lap | 6y | NM | Transfer | 3 |
| Mellow* Yellow | Working | 3y | UM | Transfer | 1 |
| Moose | Herding | 10m | UM | Transfer | 1 |
| Neo* | Bully-type | 6y | NM | Transfer | 23 |
| Pearl* | Bully-type | 1y | SF | Transfer | 5 |
| Pluto | Sporting | 6m | UM | Transfer | 1 |
| Roberta* | Bully-type | 2y | UF | Transfer | 1 |
| Sadie* | Sporting | 5y 9m | SF | Stray | 4 |
| Sam* | Hound | 1y | NM | Transfer | 19 |
| Stan | Hound | 1y | UM | Transfer | 1 |
| Thor* | Bully-type | 1y | NM | Return | 7 |
| Titus* | Bully-type | 11y | NM | Transfer | 20 |
| Wilson | Herding | 1y 6m | NM | Return | 3 |

| Pair-Housed Dogs | | | | | |
| --- | --- | --- | --- | --- | --- |
| Dog | Breed | Age | Sex | Source | Days In Shelter |
| Bluebird | Hound | 1y | SF | Transfer | 1 |
| Bo* | Working | 6m | NM | Return | 7 |
| Bobo* | Hound | 3y | NM | Transfer | 8 |
| Bugs | Herding | 10m | UF | Transfer | 1 |
| Caesar* | Sporting | 2y | NM | Transfer | 1 |
| Devin* | Lap | 7y 5m | NM | Surrender | 1 |
| Doc* | Herding | 4y | NM | Transfer | 1 |
| Goldie* | Hound | 4y | SF | Surrender | 11 |
| Henry* | Hound | 10y | NM | Return | 7 |
| Iggy* | Lap | 2y | UM | Transfer | 1 |
| Jackson * | Terrier | 2y 6m | NM | Transfer | 3 |
| Junior* | Bully-type | 1y | NM | Transfer | 5 |
| Katie | Working | 3y | SF | Transfer | 1 |
| Leela | Working | 1y | UF | Transfer | 1 |
| Lucy* | Lap | 11m | UF | Surrender | 2 |
| Maggie* | Hound | 1y | SF | Transfer | 11 |
| Mercy | Bully-type | 2y | SF | Surrender | 1 |
| Mighty* | Bully-type | 1y | NM | Transfer | 1 |
| Monkey | Herding | 10m | UM | Transfer | 1 |
| Oreo* | Bully-type | 8y | NM | Transfer | 20 |
| Reginald* | Working | 3y | UM | Transfer | 1 |
| Rex* | Terrier | 2y | UM | Transfer | 1 |
| Riley | Sporting | 2y | SF | Transfer | 1 |
| Robert | Sporting | 2y | UM | Transfer | 1 |
| Robin* | Sporting | 2y | UF | Transfer | 1 |
| Roo* | Bully-type | 2y 6m | SF | Transfer | 1 |
| Sammy | Lap | 2y | UM | Transfer | 1 |
| Sammy | Lap | 1y | UM | Transfer | 1 |
| Shadow* | Sporting | 1y 6m | UF | Stray | 8 |
| Skipper* | Bully-type | 2y 2m | UM | Transfer | 1 |
| Zuzu | Lap | 7y | NM | Surrender | 1 |

Demographic data of the enrolled dogs. Age is reported in years (y) and months (m). Sex: F is female, M is male, S is spayed, N is neutered, U is unaltered. Under breeds the predominant breed type is listed following that of Protopopova et al. [1]. * indicates dogs that we collected urine from for cortisol:creatinine analysis.

**References**

1. Protopopova, A, Gilmour, AJ, Weiss, RH, Shen, JY, Wynne, CDL. The effects of social training and other factors on adoption success of shelter dogs. Appl Anim Behav Sci. 2012;*142*(1-2):61-68.
