# Supplementary material for: Effects of single- or pair-housing on the welfare of shelter dogs: Behavioral and physiological indicators
Source: PLoS One. 2024 Jun 12;19(6):e0301137. doi: 10.1371/journal.pone.0301137 (PMC11168620; doi:10.1371/journal.pone.0301137)
Supplement: S2 Table — (DOCX) [file pone.0301137.s002.docx]

**Table S2. Demographic Distribution Across Groups**

| Intake Type | | | | |
| --- | --- | --- | --- | --- |
|  | Single-Housed | Pair-Housed | |  |
| Transfer | 17 | 23 |  |  |
| Stray | 2 | 1 |  |  |
| Surrender | 6 | 5 |  |  |
| Return | 5 | 2 |  |  |
| Breed Type | | | | |
| Bully-type | 8 | 6 |  |  |
| Herding | 5 | 3 |  |  |
| Hound | 2 | 5 |  |  |
| Lap | 5 | 7 |  |  |
| Terrier | 0 | 2 |  |  |
| Working | 5 | 4 |  |  |
| Sporting | 5 | 5 |  |  |
