# Supplementary material for: Effects of single- or pair-housing on the welfare of shelter dogs: Behavioral and physiological indicators
Source: PLoS One. 2024 Jun 12;19(6):e0301137. doi: 10.1371/journal.pone.0301137 (PMC11168620; doi:10.1371/journal.pone.0301137)
Supplement: S3 Table — (DOCX) [file pone.0301137.s003.docx]

**S3 Table. Behavioral Definitions**

| Name | | Definition | | Source |
| --- | --- | --- | --- | --- |
| Body Position | | | | |
| Front of kennel | Located between front of kennel and up to and including the midpoint of kennel. For end kennels, this also includes interacting with the side window (looking up at the window while next to it, or standing up and looking out the window). | | [1] | |
| Back of kennel | Located between back wall of kennel and up to, but not including, the midpoint of kennel | | [1] | |
| Lying down | Lying down with limbs either tucked under or placed in front of body | | [1] | |
| Sitting | Supported by two extended front legs and two flexed back legs | | [1] | |
| Standing | Supported upright with all four legs | | [1] | |
| Belly up | Lying/sitting on ground lifting hind leg, or rolling onto back exposing ventral side | | [1] | |
| Beg position | Two front paws lifted off the ground simultaneously while the back legs remain flexed | | [1] | |
| Cowering | Lying/sitting on ground lifting hind leg, or rolling onto back exposing ventral side | | [1] | |
| Play bowing | Lowered anterior and heightened posterior part (standing on hind legs) | | [1] | |
| Play bouncing | Dog repeatedly engages in a play bow behavior (>3 times) within 10 s and may bark repeatedly | | [2] | |
| Pawing at door | One front paw makes contact with the door and does not stay on the door but is moved off quickly | | [1] | |
| Head up (lying down) | Lying down on ventral or side of body with limbs either tucked or placed in front of body without head making contact with arms, paws, bed, or floor | | [3] | |
| Head down (lying down) | Lying down on ventral or side of body with limbs either tucked or placed in front of body with head resting on limbs, paws, bed, or floor | | [3] | |
| Face Orientation & Movement | | | | |
| Facing forward | Head is oriented such that an observer standing at the front of the kennel would be able to see more than the side profile of face. For end kennels, this should be considered from the perspective of the front of the kennel or from the side window. | | Adapted from [1] | |
| Facing away | Head is oriented such an observer standing at the front of the kennel would not be able to see more than the side profile of face. For end kennels, this should be considered from the perspective of the front of the kennel or from the side window. | | Adapted from [1] | |
| Gazing | Eye contact with the eyes of the observer (only for the 8:00 am videos) | | [1] | |
| Tilting head | Entire head is quickly oriented laterally and held stationary for at least 1 second | | [1] | |
| Ears back | Ears folded against sides and/or back of head and having a flattened appearance | | [1] | |
| Lip licking | Extrudes portion of tongue and runs it over its lips | | [4] | |
| Tail Position | | | | |
| Tucking tail | Tail held still and tightly between hind legs, may be curled under genital area or ventral side | | [1] | |
| Wagging tail | Tail moves perpendicularly to the dog's body | | [1] | |
| Locomotion | | | | |
| Moving forward | Distance between the dog and the front of the kennel is decreased (only code during 8:00 am videos) | | Adapted from [1] | |
| Moving away | Distance between the dog and the front of the kennel is increased (only code during 8:00 am videos) | | Adapted from [1] | |
| Standing on kennel | Both front paws make contact with the kennel and dog maintains position for >1 second (ex. Dog standing to look out window) | | Our definition | |
| Jumping on kennel | Both front paws make contact with the kennel door that does not include lunging | | [1] | |
| Lunging | Quick diagonal forward motion; may be accompanied by barking, growling, or piloerection | | [1] | |
| Pacing/circling | Repeatedly (>3 times) locomoting around kennel in fixed route completed within 20 s | | [1], [2] | |
| Chasing tail | Orients towards tail repeatedly (>3 times) and continuously | | [1] | |
| Wall bouncing | Dog repeatedly (>3 times) jumps up kennel wall from side to side | | [2] | |
| Enclosure Contact/Exploration | | | | |
| Leaning on door (sitting/standing) | Prolonged (>1 sec) contact with the kennel wall by pushing side of body against the kennel door | | Adapted from [1] | |
| Leaning on door (lying down) | Prolonged (>1 sec) contact with the kennel wall by pushing side of body against the kennel door while in a lying down position | | Adapted from [1] | |
| Chewing bedding | Dog chews its own bedding | | [2] | |
| Chewing/licking kennel | Repeatedly licks, chews, and/or bites at kennel door or wall | | Adapted from [1] | |
| Leaning on wall (sitting/standing) | Prolonged (>1 sec) contact with the kennel wall by pushing side of body against the kennel wall | | Adapted from [1] | |
| Leaning on wall (lying down) | Prolonged (>1 sec) contact with the kennel wall by pushing side of body against the kennel wall while in a lying down position | | Adapted from [1] | |
| Rubbing on kennel wall | Touches kennel wall for at least 1 second while dog walks forward. Can be scored as pacing/circling if dog does this repeatedly | | Our definition | |
| Sniffing | Muzzle/nose is oriented in a clearly observable direciton and motion of nostrils is observed | | [1] | |
| On bed | Has three or more paws on the kuranda dog bed. Any position (sitting, standing, or lying down) | | Our definition | |
| Vocalization | | | | |
| Barking | Vocalization of very short duration and low frequency | | [1] | |
| Howling | Prolonged high-amplituded vocalization of varying pitch, lips drawn together while exhaling | | [1] | |
| Whining | A cyclic vocalization | | [1] | |
| Grooming and Maintenance | | | | |
| Scratching | Paw makes repeated contact with body/face; head may be angled in direction of moving limb | | [1] | |
| Licking self | Oral contact with any part of body | | [1] | |
| Shaking off | Motions body and/or head back and forth repeatedly and rapidly | | [1] | |
| Yawning | Opens mouth widely and inhales | | [1] | |
| Stretching | Extending body and one or more front and/or hind-legs while remaining stationary | | [1] | |
| Panting | Tongue exposed with audible and/or observable breathing | | [1] | |
| Trembling | Visible shaking while dog is standing still or cowering | | [1] | |
| Regurgitating | Matter expelled from mouth with jaws open; may be preceded by repeated abdominal heaving | | [1] | |
| Eliminating | A hind-leg lifted or is squatting and urinates/defecates | | [1] | |
| Coprophagy | Feeding on own/other dogs' feces | | [1] | |
| Social Interaction & Play | | | | |
| Physical contact* | Dogs in physical contact with each other and not during a play or aggressive bout (e.g., sleeping or standing while in contact) | | Our definition | |
| Proximity* | Dogs not in physical contact but within one dog's length of the largest dog of the pair for at least 2 seconds | | Our definition | |
| Play initiation* | Play behavior from one dog (pawing at the other dog, inhibited bite, hip check) that does not result in mutual play | | Our definition | |
| Dog-dog play* | Simultaneous, reciprocal affiliation between two dogs that is not agonistic and includes at least one of the following components: self-handicapping, inhibited biting, wrestling, chasing, and exaggerated predatory behaviors. Play initiaition behaviors (e.g., play bow, pawing) from one dog were only recorded if they led to a reciprocal play bout within three seconds. | | Mehrkam (personal communication) | |
| Humping* | Grasping another dog with front legs and clasping while rest of body is arched over dog. | | Our definition | |
| Pair object play* | Same as dog play except an object (toy or bedding is involved). Usually involves tug-of-war or chase behavior. | | Mehrkam (personal communication) | |
| Single object play | Engagement with a toy or other object that involves tossing the toy, chewing or squeaking it, rolling it and chasing/pouncing on it. Object could be bedding, but excludes chewing bedding. | | Mehrkam (personal communication) | |
| Aggression | | | | |
| Growling | Throaty, rumbling vocalization; usually low in pitch | | [1] | |
| Snapping* | Teeth snap in the air at other dog but does not touch skin | | [5] | |
| Show teeth | Vertical retraction of lips to show teeth | | [5] | |
| Inhibited bite* | Makes contact with another dog with teeth but inflicts little or no damage (at most 1 to 4 punctures, with no puncture deeper than 1/2 the length of the dog's canine tooth). This excludes biting while playing. | | Adapted from [6] | |
| Uninhibited bite* | Makes contact with another dog with teeth and inflicts moderate to severe damage (at least 1 to 4 punctures with at least one puncture deeper than 1/2 the length of the dog's canine tooth) | | Adapted from [6] | |

**References**

1. Protopopova, A, Mehrkam, LR, Boggess, MM, Wynne, CDL. In-kennel behavior predicts length of stay in shelter dogs. PLoS ONE. 2014;9(12):e114319.
2. Stephen, JM, Ledger, RA. An audit of behavioral indicators of poor welfare in kenneled dogs in the United Kingdom. J App Anim Welf Sci. 2005;8:79-95.
3. Gunter, L. Understanding the impacts of breed identity, post-adoption and fostering interventions, & behavioral welfare of shelter dogs. [dissertation]. Tempe, AZ: Arizona State University; 2018.
4. Rooney, NJ, Gaines, SA, Bradshaw, JW. Behavioural and glucocorticoid responses of dogs (*Canis familiaris*) to kennelling: investigating mitigation of stress by prior habituation. Physiol Behav. 2007;92:847-854.
5. Center for Shelter Dogs. Match-Up II shelter dog rehoming program*.* Retrieved from <http://matchupiionline.centerforshelterdogs.org>
6. Association of Professional Dog Trainers. **Dr. Ian Dunbar’s dog bite scale** Available from: <http://www.dogtalk.com/BiteAssessmentScalesDunbarDTMRoss.pdf>
